# A conversation about cultivated meat

**DOI:** 10.1038/s41467-023-43984-8

**Published:** 2023-12-14

**Keywords:** Tissue engineering, Biotechnology, Sustainability

## Abstract

Summary: Cultivated or cultured meat is promising to revolutionize the food industry in the coming years to decades, helping to resolve concerns related to the environmental impact and ethical implications linked to conventional meat production. We talked to *Dr. Sandhya Sriram*, the Group CEO and Co-founder of Shiok Meats Pte. Ltd., Singapore; *Prof. Shulamit Levenberg*, the former Dean of the Biomedical Engineering Department at the Technion, current Director of the Technion Center for 3D Bioprinting and The Rina & Avner Schneur Center for Diabetes Research, as well as the Co-founder and Chief Scientific Adviser of Aleph Farms, Israel; and *Dr. Timothy Olsen*, Head of Cultured Meat in the Life Science business at Merck KGaA, Germany; about this relatively new and quickly developing sector. They explain what their teams are working on, including the biggest recent accomplishments, speak about the main challenges facing the field and how they can be resolved, and share their visions about the future of cultivated meat, aiming to provide more equitable and sustainable access to nutritious food for the growing world population.

**Figure:**
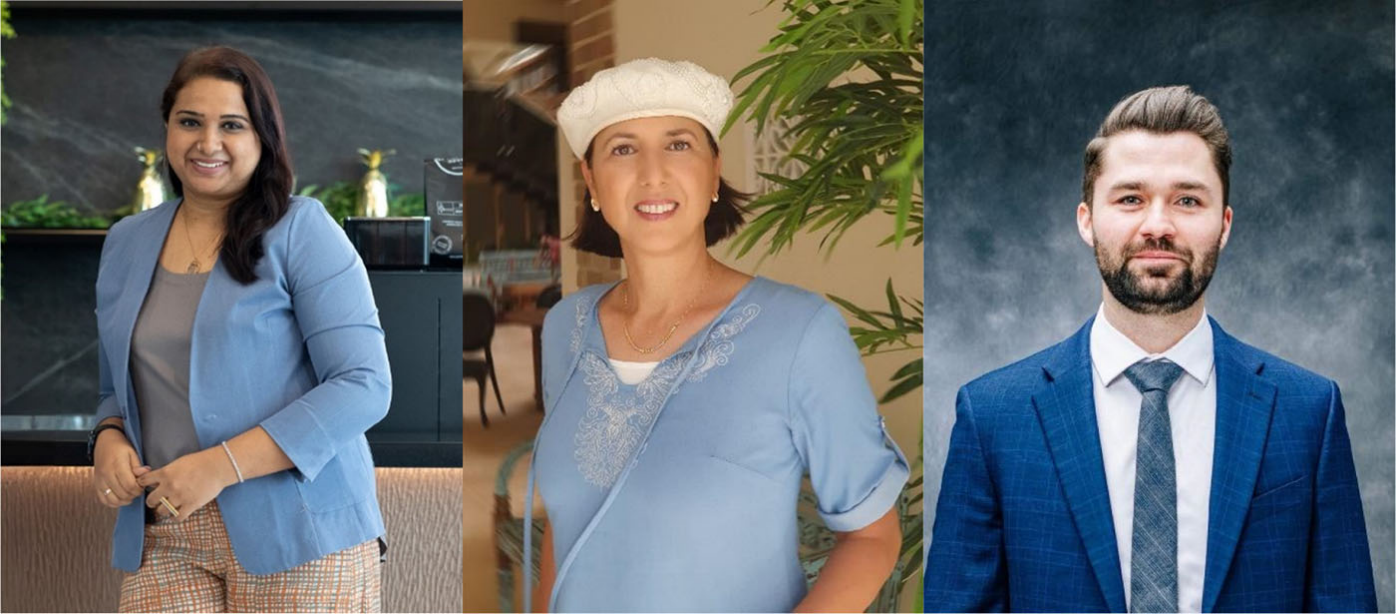
Sandhya Sriram from Shiok Meats (left), Shulamit Levenberg from the Technion and Aleph Farms (middle), Timothy Olsen from Merck (right). Shiok Meats, Revital Tubul, Timothy Olsen from left to right.

We interview Sandhya Sriram (SS) from Shiok Meats, Shulamit Levenberg (SL) from the Technion and Aleph Farms and Timothy Olsen (TO) from Merck on the current status, challenges and future directions of cultured meat.

Please briefly describe what your team is currently working on.

**SS:** Shiok Meats, established in 2018, is a cultivated, cell-based meat and seafood company, headquartered in Singapore. It is the first of its kind in Singapore and South-East Asia with a mission to bring delicious, sustainable and healthy shrimp, lobster, crab and meats to your table, by harvesting from cells instead of animals. Our meats are animal-, health- and environment-friendly with similar taste and texture compared to their traditional counterparts. Shiok Meats also own Southeast Asia’s first cultivated red meat company, Gaia Foods.

We currently have two sub-teams working on 1) establishing stable cell lines for cultivated shrimp and lobster meat production, so that we can scale up and 2) scaling up cultivated beef production. Both projects involve gathering enough experimental data for the respective regulatory approvals.

**SL:** Aleph Farms’ primary focus right now is on commercializing cultivated beef steaks grown from non-modified cow cells, and on developing other animal products, such as collagen, from those same cells. Our first product—which falls under our recently announced product brand, Aleph Cuts—is a cultivated thin-cut beef steak called the Petit Steak. We plan to introduce the Petit Steak to diners in Singapore and Israel this year upon receiving regulatory approvals. We are also working on producing thicker, fattier cuts of cultivated beef to meet diverse consumer preferences around the world.

For cellular agriculture to achieve scale and impact, Aleph Farms is taking a leading role in building, from the ground up, a resilient supply chain and set of infrastructures suitable for large-scale production. It is also working alongside the public sector to shape policies, harmonize regulatory pathways and lead an inclusive and just transition to more sustainable and equitable food systems.

**TO:** At Merck, a multidisciplinary team is responsible for delivering industry-enabling technologies, products, and services for the safe and scaled production of cultured meat—from R&D to pilot scale, and commercial manufacturing. The topic directly aligns with our corporate sustainability goals and existing core strengths developed in our Life Sciences business unit (known in the U.S. and Canada as MilliporeSigma). Our flagship program builds and delivers scalable, cost-effective cell culture media solutions with appropriate regulatory documentation. A second R&D program leverages our company’s expertise in membrane and filtration technologies to focus on meeting the consumer demand for higher-value structured and differentiated whole cut meats via a hollow fiber bioreactor system composed of edible and food safe materials. Our technology roadmap includes developing fit-for-purpose solutions based on industry needs in media, cells, scaffolds, biomonitoring, safety testing, and upstream/downstream manufacturing unit operations.

Looking back at the past 5 years, could you tell us about the biggest accomplishment that your team has achieved?

**SS:** Being the first company to work on cellular agriculture in Singapore and Southeast Asia, I would say that our biggest achievement has been educating the industry and consumers on various aspects of this technology and sector. This whole cultivated meat/seafood industry is less than a decade old and much younger in the Asia Pacific region, so it has been a lot of educating and learning for us, as well as the consumers on this new technology and the diverse product palette (Fig. 1).

**Fig. 1:**
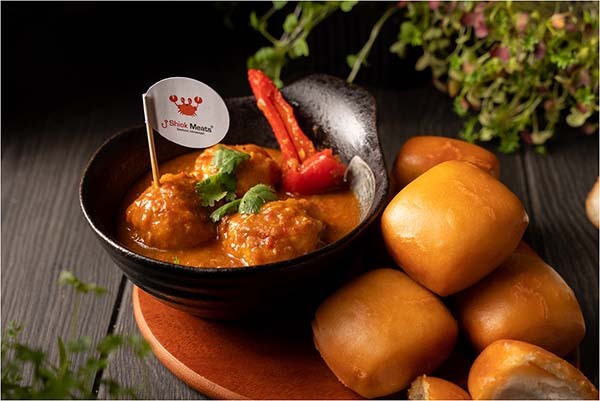
Shiok chilli crab dish (image credit Shiok Meats).

**SL:** It’s hard to select just one! Three highlights would be revealing the world’s first cultivated thin-cut beef steak in 2018 (the basis for our Petit Steak), announcing the world’s first cultivated ribeye steak in 2021, and becoming the first company to receive a kosher ruling for cultivated beef from Israel’s Chief Rabbi in early 2023.

We anticipate becoming the world’s first company to have a cultivated beef steak approved for sale in Singapore and Israel later this year, pending respective regulatory approvals. We are working closely with regulators in both countries and look forward to introducing diners to our “new take on steak.”

**TO:** Over the last 5 years, our team has worked hard to build relationships with cultured meat startups, key academic institutions, regulatory agencies, nonprofit think tanks, and other multinational corporations to learn about and work together to shape the new and developing cultured meat industry. As our company had existing expertise in cell culture media development and manufacturing for the pharma industry, it was critical that our team understood the industry-specific challenges and requirements of cultured meat companies, so that we could address these with our company’s strengths and core competencies. After extensive discussions with industry representatives, it was clear that our customers don’t just need our company to produce cell culture media products that work for today, but they need a partner that will commit to develop more affordable cell culture media over time and include industry-leading quality documentation. Providing exactly this to our customers in early product development efforts is leading to the acceleration of timelines for cultured meat products to come to market. These results are due to our team’s global presence, thought leadership, demonstrated technical expertise, customer-centric approach, and ultimately taking action to have impact—and I would that say this is the biggest accomplishment that our team has achieved.

What do you think are the main challenges facing the field of cultured meat?

**SS:** I see four main challenges that I would like to elaborate on.

· **Cost:** Currently, cultivated meat is much more expensive than traditional meat, given how complex the manufacturing technology is.

· **Scalability:** Startups in this space are working tirelessly to go beyond the production in small batches and reach pilot/commercial scale over the next few years, which will help meet the demand once products hit the market. Larger scales will reduce costs and product pricing.

· **Regulatory approval**: More countries beyond Singapore and the USA need to develop comprehensive regulatory frameworks for cultivated meat. This will help expand the footprint of companies beyond these two markets.

· **Acceptance:** Consumers’ acceptance depends mainly on how we educate them, which is why, as an industry, we need to consciously work on openly and transparently sharing the benefits of cultivated meat and address any concerns consumers may have about its safety.

**SL:** Challenges in the cultivated meat space that will need to be overcome for this technology to reach its full potential include overcoming at-scale production costs and developing the right products to meet consumer acceptance. We see consumer acceptance heading in the right direction very quickly. The more that people come to understand what cultivated meat entails (and doesn’t entail), the more they see it as a safe source of high-quality protein. Research from across different markets shows that many people are actually quite eager to try cultivated meat. This is one of the reasons that we are very confident about the prospects of long-term consumer acceptance. We acknowledge that to turn this expected acceptance into actual consumption patterns over time, it is crucial that we develop the right products, which means developing and using the right technology.

**TO:** Two of the biggest challenges today are centered around reducing costs and scaling up production, both of which will be required for successful commercialization and mass adoption by the consumer. A tremendous amount of development effort is focused on improving cell and media productivities, which will lead to improved bioprocess yields, and ultimately reduce manufacturing operational costs (Fig. 2).

**Fig. 2:**
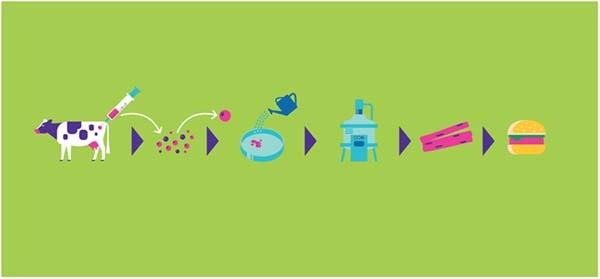
Simplified workflow for cultivated meat production (image credit Merck).

Economies of scale in purpose-built cultured meat manufacturing facilities will help realize cost savings and lessons from large food companies can be adopted. Final product formulation can also help address costs. Hybrid products are a combination of plant-based protein with a varying percentage of cultured meat. The hybrid approach will likely be used by companies to bring their “Product 1.0” to market. By not having 100% cultured products, final product costs can be reduced and allow companies to compensate for the limited available cultured meat manufacturing capacity available at this time. Beyond cost and scale challenges, we must remember that the consumer is the ultimate decision-maker. Early consumer data reports summarized in the Good Food Institute 2022 State of the Industry Report for Cultivated Meat and Seafood are indicating that about 2/3 of consumers are willing to try cultured meat, a great indicator for an industry in its infancy.

What do you think is the most important of these challenges, and how does your team plan to solve it?

**SS:** All of these challenges go hand in hand, so they continue to be our priorities in the coming months.

Scale up is a definite challenge—we are learning along the way as there is no playbook for this technology. The cell culture media contributes to the majority of the cost for cultivated meat. We have switched this media from pharma-grade to food-grade, which has already brought the cost down significantly, but this remains an ongoing process. Food-grade media is an important aspect of cultivated meat as every ingredient used in the process of producing the meat needs to be edible and safe for consumption. As for seeking regulatory approval in more countries, we are actively speaking with consultants and local regulatory authorities to prepare a comprehensive dossier for submission.

Consumer education is an always-on activity at Shiok Meats through outreach events, internships, food fairs, and thought leadership.

**SL:** Reducing at-scale production costs is one primary challenge for the industry. For widespread consumer adoption to happen, companies need to produce enough cultivated meat in a cost-efficient way to satisfy demand and curiosity.

At Aleph Farms, we’re taking a multifaceted approach to overcoming at-scale production costs:

- We establish strategic supply chain agreements that help keep raw materials costs in check. Recently, we announced the latest such agreement with Thermo Fisher Scientific for the production of growth media at the quantity, quality and cost that match our scale-up and cost reduction strategies.

- As part of our inclusive leadership model, Aleph Farms is committed to supporting the wide spectrum of companies that produce cultivated meat. We extend this sector-supporting leadership role to some of our supply chain agreements by establishing them as open agreements. As a result of such agreements, any company that produces cultivated meat can access affordable proteins and avoid the need to resort to fetal bovine serum or animal-derived ingredients. This inclusive approach lowers raw material costs and drives economies of scale for the cultivated meat space at large.

- We are also working with key food industry partners to expand our production and commercialization capabilities. These partnerships allow us to leverage the expertise and capabilities of leading food and meat companies like Cargill (US), Migros (Switzerland), BRF (Brazil), Thai Union (Thailand), Mitsubishi Corporation (Japan) and CJ CheilJedang (South Korea).

- We continually innovate within our production process to help ensure that we can produce cultivated meat as efficiently as possible. This includes developing specific technological modules geared towards maximizing efficiency.

- Our growth strategy involves increasing production capabilities while keeping capital expenditures lean by directing a significant portion of those expenditures to existing infrastructure. Nimble capital deployment focused on smaller-scale facilities in our initial markets lowers risk and allows us to bridge capacity gaps before becoming fully operational in additional markets. For example, in March 2023, we acquired a manufacturing facility in Modi’in, Israel and certain related assets from the biotechnology company VBL Therapeutics. We also signed a memorandum of understanding with ESCO Aster, a vertically integrated contract manufacturing organization, to produce cultivated meat in Singapore.

**TO:** Cell culture media is a key raw material input for all cultured meat companies, and it accounts for about 50% of material costs today. To achieve commercialization at scale, media must be cost-efficient, produced from a robust supply chain of raw materials, suitable for effective growth and differentiation into specific cell types, free of any animal-derived materials, and produced in a facility with an appropriate quality management system. Our cell culture media team leverages our company’s existing expertise and feedback from customers to provide cost-effective and scalable solutions that meet the quality, regulatory and performance needs of the industry. Our efforts are focused on having impact on basal media to start, which is composed of the salts, sugars, vitamins, and amino acids required by all cells. In large-scale production of dry powdered basal media, raw materials dominate the costs. Our goal is addressing these cost challenges by building a cultured meat cell culture media raw material supply chain composed of pharma and food grade raw materials that enable the cost-effective production of any basal media formulation at scale with the appropriate supporting quality and regulatory documentations. The fit-for-purpose raw material supply chain will balance performance, quality and costs, while leveraging efficiencies provided with scaled manufacturing, which will allow our company to pass savings on to the customer. We are currently providing dry powdered basal media on the order of tons to leading startups around the world that are building pilot-scale manufacturing facilities and bringing the first products to the market.

Looking forward to the next 5 years and considering accessibility, who do you think the main consumers of cultured meat will be? Can we expect it to be more than a niche product for well-off consumers?

**SS:** As we scale up production over the next five years and bring the cost further down, we aim to reach a more significant share of the flexitarians and meat lovers who are willing to switch to such alternatives, provided the price appeals to them. So, yes, we aim to broaden our customer base by decreasing prices. Also, our products will undergo multiple iterations, as any food product does – be it taste, nutritional content, texture, mouth-feel and so on.

**SL:** We believe in creating a high impact while enabling systemic change in our food systems. That means producing cultivated meat that is accessible to more than just the most well-off consumers. As we plan for launches in Singapore and Israel, and as we look forward to subsequent launches in additional markets, we are working with chefs, restaurants, and hospitality groups who can enable more diners to try our cultivated meat products (Fig. 3).

**Fig. 3:**
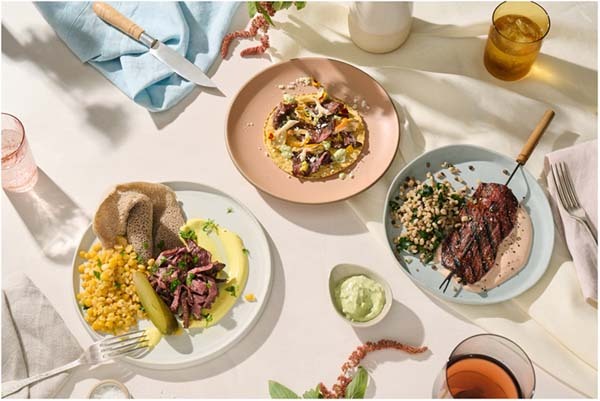
Sample dishes prepared with Aleph Cut products (image credit: Aleph Farms).

As we build up our production capabilities and supply chains, we hope to achieve price parity with the beef market within a few years of our launch.

**TO:** Over the next 5 years, it is becoming clearer that cultured meat companies will be launching products via high-end dining environments, like Michelin Star-awarded restaurants, based on the recent commercial launches from UPSIDE Foods and GOOD Meat. This approach de-risks the go-to-market in a few ways. First, it ensures that the initial cultured meat and seafood products that consumers experience are prepared with the highest quality and consistency in presentation, thereby ensuring a great first impression with the consumer. Second, high end restaurants offer opportunity for press and news outlets to report on the industry, which can help educate all consumers. Studies have been showing that consumers want to be informed of what they are eating, and willingness to try cultured meat increases as more background information is provided. Third, there simply is not enough capacity to produce the volumes of cultured meat required to reach every consumer today. Catering to high end restaurants and commercializing cultured meat as a niche product for the next 5 years will provide the opportunity for early consumer testing, while the cost and scale challenges continue to be addressed by cultured meat startups. I think significant progress can be made over the next 5 years for these challenges with continued funding, advancements in technology, expanding capacity with new facilities and leveraging contract manufacturing organizations, securing regulatory approvals, and increasing government involvement. Given this, I project an inflection point in the mid-2030s that will allow cultured meat to be more accessible in food service environments, like restaurants that the average consumer frequents, and in some higher-end grocery stores. As we approach 2040 and beyond, I think we will be able to purchase many cultured meat product types at grocery stores with prices comparable to conventional meat products, which will allow these products to be purchased by the average consumer on a regular basis.

Do you see plant-based alternatives to be complementary to or competing with cultured meat?

**SS:** Complementary, definitely. Plant-based alternatives and cultivated meat are both responses to traditional meat production’s environmental impact and ethical implications. It is up to the consumer to choose which alternative works best for them in terms of taste, texture, price, and sustainability quotient. In fact, most of our and other cultivated meat company products are hybrid blends with plant-based ingredients.

**SL:** Complementary, certainly. Part of our inclusive approach is acknowledging the fact that there are—and will continue to be—multiple solutions that help make food systems more diverse, secure and resilient. Global demand for quality protein is on the rise, so there is most likely a solid long-term place in the market for plant-based meat. Still, we believe that animal cells – as a new category of animal products—are particularly strategic in terms of providing diners with what they want: new and exciting choices that don’t compromise on quality.

**TO:** We need to build a food system to sustainably produce protein for the next billion people, as we approach a population of 10 billion by 2050, while helping to positively impact climate, animal welfare, and our oceans. There is not one simple solution to this huge challenge. Our company views cultured meat as one approach to feed the world sustainably and actively supports plant and fermentation-based production, sustainable farming technologies, and other initiatives that use science as a force for good to disrupt the food system.

